# Diagnostic Test of *Transforming Growth Factor-Beta* 1 (TGF-*β*1) in Menstrual Blood with Endometriosis

**DOI:** 10.1155/2023/9970818

**Published:** 2023-12-11

**Authors:** Kemas Yusuf Effendi, Excellena Nasrul, Iskandar Zulqarnain, Theodorus, Rizani Amran, Heriyadi Manan, Adnan Abadi, Fatimah Usman, Cindy Kesty

**Affiliations:** ^1^Division of Reproductive, Endocrinology, and Infertility, Department of Obstetrics and Gynecology, Dr. Mohammad Hoesin General Hospital, Faculty of Medicine, Universitas Sriwijaya, Palembang, South Sumatera, Indonesia; ^2^Department of Obstetrics and Gynecology, Dr. Mohammad Hoesin General Hospital, Faculty of Medicine, Universitas Sriwijaya, Palembang, South Sumatera, Indonesia; ^3^Medical and Health Research Unit, Faculty of Medicine, Universitas Sriwijaya, Palembang, South Sumatera, Indonesia

## Abstract

**Background:**

Endometriosis is a benign disorder that is generally defined as the presence of endometrial glands and stroma outside their normal location. TGF-*β*1 is found in stromal cells and its expression is increased in epithelial cells of endometriotic cysts. Endometriosis diagnostics take a long time, so new markers are needed to diagnose endometriosis. This study aims to determine the diagnostic value of TGF-*β*1 in menstrual blood in diagnosing endometriosis.

**Method:**

Diagnostic tests to compare eutopic endometrial TGF-*β*1 levels from menstrual blood of patients with suspected endometriosis were undertaken in the Obstetrics and Gynecology Department of Dr. Mohammad Hoesin General Hospital, Faculty of Medicine, Sriwijaya University, Palembang, from July 2019 to November 2020. 50 patients who were suspected with endometriosis met the inclusion criteria. Comparison of TGF-*β*1 levels between endometriosis and nonendometriosis patients was analyzed using the Mann–Whitney test. The cutoff point of the TGF-*β*1 level towards the histopathological outcome was obtained using the ROC curve. Data analysis was performed by using SPSS version 22.0.

**Results:**

In this study, endometriosis patients were 31.6 ± 6.55 years of age with a range of 20 to 46 years. In statistical analysis, there was no difference in BMI (*p* = 0.181) and BMI classification (*p* = 0.207), the history of contraception (*p* = 0.097), infertility (*p* = 1.000), and dysmenorrhoea (*p* = 1.000) between endometriosis and nonendometriosis patients. In the study, there were differences in TGF-*β*1 between endometriosis and nonendometriosis patients (*p* ≤ 0.001). By using the ROC curve, the cutoff point for TGF-*β*1 levels has the best sensitivity and specificity, which is 515 ng/ml. The TGF-*β*1 level has a sensitivity of 80%, a specificity of 90%, a positive predictive value (PPV) of 0.969, a negative predictive value (NPV) of 0.529, a positive likelihood ratio of 8, a negative likelihood ratio of 0.222, and an accuracy of 0.820 to the endometriosis outcome.

**Conclusion:**

It can be concluded that the TGF-*β*1 level has a very good diagnostic value in establishing endometriosis diagnostics. This trial is registered with ISRCTN72218532.

## 1. Introduction

Endometriosis is the presence of endometrial glands and stroma outside of their normal location. However, in those who are refractory to medical management, surgery may be necessary [1]. It happens for about 6–10% of the general female population and 35–50% of patients experiencing pain and/or infertility [2]. Endometriosis is estimated to occur in 3–10% of reproductive aged women (ages 15–44 years), 25–35% in infertile women, 1-2% in women who undergo sterilization, 10% in hysterectomy surgery, 16–31% in laparoscopy, and 53% occur in women with severe pelvic pain who requires surgical evaluation [3].

John Sampson's hypothesis in 1920 about retrograde menstruation which causes the implantation of endometriotic tissue outside the uterine cavity was a hypothesis that was still widely accepted [4]. Another hypothesis is the lymphatic or hematogenous spread of endometrial cells. Endometrium acts similar to neoplastic cells, invading blood and lymphatic vessels and spreading elsewhere. Meyer's hypothesis in 1919 about the celomic metaplasia stated that the original celomic membrane undergoes metaplasia forming typical endometrial glands and stroma, perhaps under influence of environmental factors. Moreover, the cause of endometriosis is the dislocation of primitive endometrial tissue outside the uterine cavity during organogenesis [4]. Endometriosis also appears to be associated with elevated levels of different microorganisms across various microbiome sites. There is some scientific proof to state that the immune response may be modulated by the microbiome [5].

TGF-*β* is involved in gene expression, cell motility, proliferation, apoptosis, differentiation, immune response, and tumorigenesis [6]. In mammals, three types of TGF-*β*, namely, TGF-*β*1, TGF-*β*2, and TGF-*β*3, have been cloned and proven to have overlapped in vitro functions [7]. Secretion of TGF-*β* into the peritoneal fluid of women with endometriosis suggests that TGF-*β* may be important in the formation and/or development of endometriosis [2].

The diagnosis of endometriosis is reached by laparoscopic intervention followed by histological confirmation of ectopic endometrial glands and stroma. Because of the variability of symptoms and confusion by other disorders, the diagnostic period for endometriosis is long, about 6–9 years [8]. The new approach to TGF-*β*1 may be of interest as not many reports have been published on this subject. D'Hooghe et al.'s study did not show an association between higher serum TGF-*β*1 levels and higher stages [9]. Meanwhile, Pizzo et al.'s study explained that higher levels of TGF-*β*1 in peritoneal fluid were associated with higher stage specificity in endometriosis [10]. There are no studies that discuss the relationship between TGF-*β*1 levels in menstrual blood and endometriosis. Thus, this study aims to determine the levels of transforming growth factor-beta 1 (TGF-*β*1) in menstrual blood as an angiogenic factor to the degree of endometriosis.

## 2. Method

This study is a clinical trial without comparison. The research was conducted at the Department of Obstetrics and Gynecology, Dr. Mohammad Hoesin General Hospital Palembang. Data collection and observation were carried out from July 2019 to December 2019. The sample was all patients with suspected endometriosis who underwent laparoscopy and met the inclusion criteria. Patients who agreed to participate in this study signed a consent form.

Patients with suspected endometriosis who underwent laparoscopy with an age range of 25–35 years and >35 years until before menopause, married, agreed to undergo operative laparoscopy, agreed to participate in the study, and signed an informed consent were included in this study. Patients with suspected malignancy, chronic inflammation, diabetic retinopathy, pregnancy, and use of GnRH agonists before laparoscopy were excluded in this study. In addition, the criteria for dropout in this study were patients with blood cells lysis on examination. Withdrawal criteria in this study were subjects who refused to continue the study. Samples were collected by means of consecutive sampling. In this study, 58 people who met the inclusion criteria were found, and 8 people dropped out. The total samples were 50 patients.

All patients who will be included in this study have anamnesis (name, age, address, parity, last menstrual period (LMP), previous medical history, previous use of contraception, and history of drug use) and physical examination (general condition, blood pressure, pulse, respiration rate, temperature, height, and weight). Menstrual blood samples were collected before surgery when the patient was menstruating for the first three days and obtained through storage in the menstrual cup. Menstrual blood samples were immediately taken to the laboratory and tested for TGF-*β*1 expression by the quantitative sandwich enzyme immunoassay method. Prepare the reagents and samples, enter 50 L of assay diluent RD1-73 into each well (plate has been coated with anti-TGF-*β*1), and add 50 L of standard, control, and activated sample to each well containing the assay diluent. Then, incubate for 2 hours, rinse and wash 4 times using a washing buffer, add 100 L of conjugate and incubate again for 2 hours, rinse and wash 4 times using a washing buffer, and add 100 L of substrate solution and incubate for 30 minutes. Keep away from light, add 100 L of the reaction-stopping solution, and read at a wavelength of 450 nm for 30 minutes. The correction wavelength is 540 or 570 nm. Plot the standard curve and estimate the sample concentration against the curve.

Then, laparoscopy was performed by a reproductive endocrinology in fertility consultant to confirm the diagnosis and degree of endometriosis. The intraoperative findings were followed by a histopathological examination to confirm the diagnosis of endometriosis. After the data were collected, statistical analysis was carried out. Comparison of TGF-*β*1 levels between endometriosis and nonendometriosis patients was analyzed using the Mann–Whitney test. The intersection point of the TGF-*β*1 level towards the histopathological outcome was obtained using the ROC curve. Data analysis was performed by using SPSS version 22.0. Data were presented in tables and curves in order to facilitate data reading and result analysis.

## 3. Result

From 50 samples, 40 subjects have endometriosis (first degree 1 subject, second degree 3 subjects, third degree 6 subjects, and fourth degree 30 subjects). Ten subjects have not endometriosis. The general characteristics of the study subjects are shown in Table 1. Endometriosis patients have a mean age of 31.6 ± 6.55 years with an age range of 20 to 46 years, and the most age classification is 20–35 years (70%). Meanwhile, nonendometriosis patients have a mean age of 35.1 ± 7.03 years with an age range of 26 to 48 years and the most age classification is 20–35 years (60%). With statistical analysis, it was found that there was no difference in age (*p* = 0.096) and age category (*p* = 0.707) between endometriosis and nonendometriosis patients.

In this study, endometriosis patients had a mean body mass index (BMI) of 23.13 ± 2.99 kg/m^2^ with a BMI range of 16.73 to 29.30 kg/m^2^, and the highest BMI classification was normoweight (60%). The nonendometriosis patients had a mean BMI of 24.64 ± 3.73 kg/m^2^ with a BMI range of 20.81 to 32.89 kg/m^2^, and the highest BMI classification is also normoweight (60%). By statistical analysis, it was found that there was no difference in BMI (*p*=0.181) and BMI classification (*p*=0.207) between endometriosis and nonendometriosis patients.

The majority of endometriosis patients do not use contraception (85%), suffer from infertility (65%), and have dysmenorrhoea (92.5%). Also, in nonendometriosis patients, the majority of them do not use contraception (60%), suffer from infertility (65%), and have complaints of dysmenorrhoea (100%). With statistical analysis, it was found that there was no difference in contraceptive history (*p* = 0.097), infertility (*p* = 1.000), and dysmenorrhoea complaints (*p* = 1.000) between endometriosis and nonendometriosis patients.

In this study, the mean TGF-*β*1 level of endometriosis patients was 656.6 ± 164.92 ng/ml with a range of 97 to 982 ng/ml while nonendometriosis patients had a mean TGF-*β*1 of 163.5 ± 166.36 ng/ml with a range of 32 to 553 ng/ml. By statistical analysis, it was found that there was a difference in TGF-*β*1 between endometriosis and nonendometriosis patients (*p* ≤ 0.001) (Table 2).

**Figure 1:**
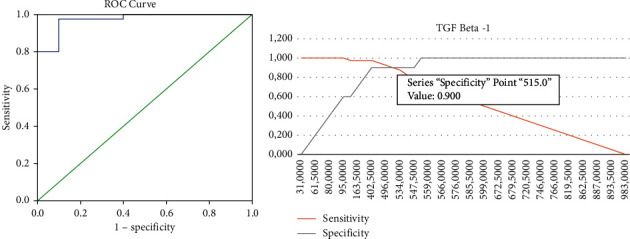
ROC of TGF-*β*1 based on laparoscopy for endometriosis.

Analyzes were performed with the ROC (Receiver Operating Curve) to find the TGF-*β*1 cutoff point to obtain the sensitivity and specificity values of TGF-*β*1 diagnostics. The ROC of the patient's TGF-*β*1 diagnostic value was based on the laparoscopy outcome of endometriosis (Figure 1). Determination of the cutoff point for the diagnostic value of TGF-*β*1 was carried out by making a curve between the sensitivity, specificity, and TGF-*β*1 levels of patients with suspected endometriosis. From the figure, it is found that the value that has the best sensitivity and specificity was 515 ng/ml.

In this study, there were 32 endometriosis patients and 1 nonendometriosis patients with TGF-*β*1 level >515 ng/mL. There were 8 endometriosis patients and 9 nonendometriosis patients with TGF-*β*1 level <515 ng/mL (Table 3).

Based on Table 4, the diagnostic value of TGF-*β*1 on the laparoscopy outcome of endometriosis has a sensitivity of 80%, a specificity of 90%, a Positive Predictive Value (PPV) of 0.969, a Negative Predictive Value (NPV) of 0.529, a positive likelihood ratio of 8, and a negative likelihood ratio of 0.222. The accuracy of TGF-*β*1 levels against the laparoscopy outcome was 0.820, which means that the degree of suitability of measurement (reliability) was very good.

## 4. Discussion

In 1999, Treloar's work on an Australian population demonstrated that genetic influence is responsible for 51% of endometriosis [11]. Angioni's study was to evaluate the influence of polymorphisms of the WNT4, VEZT, and FSHB genes, known to be involved in molecular mechanisms associated with proliferation and development of endometriotic lesions in a particular Mediterranean population, the Sardinian population. However, an analysis of recent publications on the genetics of endometriosis showed a discrepancy in the results obtained in different populations [12].

Murgia's study showed an increase in *β*-hydroxybutyric acid and glutamine and a decrease in tryptophan as well as an alteration of pathways such as nitrogen metabolism, pyrimidine metabolism, glutamine and glutamate metabolism, and aminoacyl-tRNA biosynthesis. Endometriosis can be considered an inflammatory disease with evidence of elevated levels of peritoneal fluid cytokines and growth factors, alterations in B cell activity, and an increased incidence of autoantibodies [13]. Another study explained a modification of small bowel permeability could be involved in the pathophysiology of endometriosis and the maintenance of low-grade inflammation [14].

Transforming growth factor-*β* (TGF-*β*) is a pleiotropic polypeptide, which regulates various biological processes, including embryonic development, adult stem cell differentiation, immune regulation, wound healing, and inflammation. The TGF-*β* superfamily comprises more than 35 members in vertebrates, including TGF-*β*1 [15]. To date, many cytokines thought to be involved in endometriosis have been analyzed. TGF-*β* is involved in gene expression, cell motility, proliferation, apoptosis, differentiation, immune response, and tumorigenesis [6]. In mammals, three types of TGF-*β*, namely, TGF-*β*1, TGF-*β*2, and TGF-*β*3, have been cloned and shown to have overlapping in vitro functions [7]. Secretion of TGF-*β* into the peritoneal fluid of women with endometriosis indicates that TGF-*β* may be important in the formation and/or progress of endometriosis. TGF-*β*1 is found in stromal cells, and its expression is increased in the epithelial cells of endometriotic cysts [2]. Higher TGF-*β*1 levels are associated with higher stage specificity in endometriosis [16]. Two other studies showed that subjects with endometriosis exhibited higher TGF-*β*1 levels in peritoneal fluid [2].

A positive likelihood ratio of 8 and a negative likelihood ratio of 0.222 means that the likelihood of a TGF-*β*1 ≥ 515 ng/ml level is 8 times in patients with endometriosis and a possible TGF-*β*1 level <515 ng/ml is 0.222 times in nonendometriosis patients. A positive likelihood ratio of 8 and a negative likelihood ratio of 0.222 indicate a very good impact of TGF-*β*1 on the diagnosis of endometriosis. In addition, the accuracy of TGF-*β*1 against the laparoscopic outcome of endometriosis in this study was 0.820, which means that the degree of suitability of measurement (reliability) is very good. The high sensitivity, specificity, and accuracy values in this study indicate that TGF-*β*1 can be considered as a diagnostic tool for endometriosis.

## 5. Conclusion

There was a significant difference in TGF-*β*1 levels between endometriosis and nonendometriosis patients (*p* ≤ 0.001). The cutoff point for the TGF-*β*1 level which had the best sensitivity and specificity was 515 ng/ml. The TGF-*β*1 level has a sensitivity of 80%, a specificity of 90%, a positive predictive value (PPV) of 0.969, a negative predictive value (NPV) of 0.529, a positive likelihood ratio of 8, a negative likelihood ratio of 0.222, and an accuracy of 0.820 towards the endometriosis outcome.

## 6. Suggestion

The level of TGF-*β*1 can be used as a consideration for diagnostic tools for endometriosis. Continuous research can be conducted to strengthen the results of this study.

## Figures and Tables

**Table 1:** The general characteristics of the study subjects.

Variable	Endometriosis (*n* = 40)	Nonendometriosis (*n* = 10)	*p* value
Age (year)			
Mean ± SD	31.6 ± 6.55	35.1 ± 7.03	0.096^a^
Median (min–max)	29 (20–46)	33 (26–48)	
Age category, *n* (%)			
20–35 years	28 (70)	6 (60)	0.707^b^
>35 years	12 (30)	4 (40)	
BMI (kg/m^2^)			
Mean ± SD	23.13 ± 2.99	24.64 ± 3.73	0.181^c^
Median (min–max)	23.09 (16.73–29.30)	24.01 (20.81–32.89)	
BMI classification, *n* (%)			
Underweight	2 (5)	0 (0)	0.207^d^
Normoweight	24 (60)	6 (60)	
Overweight	14 (35)	3 (30)	
Obese I	0 (0)	1 (10)	
Contraception usage, *n* (%)			
Yes	6 (15.0)	4 (40)	0.097^b^
No	34 (85.0)	6 (60)	
Infertility, *n* (%)			
Yes	26 (65)	6 (60)	1.000^b^
No	14 (35)	4 (40)	
Dysmenorrhoea, *n* (%)			
Yes	37 (92.5)	10 (100)	1.000^b^
No	3 (7.5)	0 (0)	

Note: ^a^Mann–Whitney test, *p*=0.05. ^b^Fisher exact test, *p*=0.05. ^c^Independent *T* test, *p*=0.05. ^d^Pearson chi square test, *p*=0.05.

**Table 2:** TGF-*β*1 level based on laparoscopy (*n* = 50).

Variable	Endometriosis (*n* = 40)	Nonendometriosis (*n* = 10)	*p* value
TGF-*β*1			
Mean ± SD	656.6 ± 164.92	163.5 ± 166.36	≤0.001^a^
Median (min–max)	599 (97–982)	92.5 (32–553)	

Note: ^a^Mann–Whitney test, *p*=0.05.

**Table 3:** Diagnostic test of TGF-*β*1 based on laparoscopy for endometriosis.

Levels		Laparoscopy outcome		Total
		Endometriosis	Nonendometriosis	
TGF-*β*1	≥515 ng/ml	32	1	33
	<515 ng/ml	8	9	17

Total		40	10	50

**Table 4:** Diagnostic value of TGF-*β*1 based on laparoscopic outcome of endometriosis.

Diagnostic value	
Sensitivity	90%
Specificity	80%
Positive predictive value	0.969
Negative predictive value	0.529
Positive likelihood ratio	8
Negative likelihood ratio	0.222
Accuracy	0.820
AUC	0.973 (CI 95% 0.928–1.000)

## Data Availability

We thanked all the researchers involved in this study and whose articles were included. Thanks to dr. Mohammad Hoesin General Hospital and Faculty of Medicine, Universitas Sriwijaya, Palembang, South Sumatera for helping us conceive the study.
